# Acute endocarditis in intravenous drug users: a case report and literature review

**DOI:** 10.3402/jchimp.v2i1.11513

**Published:** 2012-04-30

**Authors:** Yan Ji, Lara Kujtan, Dawn Kershner

**Affiliations:** 1 Department of Internal Medicine, Union Memorial Hospital, Baltimore, MD, USA; 2 School of Medicine, Saba University, Saba, Dutch Caribbean; 3 Department of Cardiology, Union Memorial Hospital, Baltimore, MD, USA

**Keywords:** Infective endocarditis, intravenous drug use, MSSA, TEE, Janeway lesions, subungual splinter hemorrhages

## Abstract

Infective endocarditis (IE) is a notorious complication of intravenous drug use (IDU). It typically affects the cardiac valves. Among these, the tricuspid is the most common affected valve, although the mitral and/or aortic valves can also be involved. Methicillin sensitive staphylococcus aureus (MSSA) is the most common etiological microbial agent of IE in IDU. Once IE is diagnosed, antibiotic treatment should start immediately after blood cultures have been obtained. However, IE in this particular patient population is more difficult to treat, and has a high recurrence rate compared to other patient populations, because of continuing IDU and medical non-compliance. Here, we present an interesting case of IE in a relatively young IDU patient with severe MSSA positive sepsis. The updated diagnostic and treatment strategies, as well as the ethical issues involved in the management of IE patients in the setting of current active IDU will also be discussed.

Despite rapid development of advanced diagnostic methods, antimicrobial agents and modern surgical devices, the incidence of IE has remained almost unchanged over the past few decades (1). In developed countries, however, rheumatic disease is no longer the main underlying disease. Instead, degenerative valve disease of the elderly, mitral valve prolapse and intravenous drug use (IDU) are becoming the leading causes (2, 3). IDU is the predominant cause of IE, especially in urban areas and among patients of relatively young age. The incidence of IE among IDU in the United States ranges between 1–5% every year. In IDU patients, IE accounts for 5–20% of hospitalizations and 5–10% of total deaths (4, 5).

Historically, streptococci viridans was the most common pathogen that caused native valve IE. However, MSSA has become the leading etiological agent, especially in IDU-associated IE. Moreover, infections with MRSA, enterococci or other streptococcus species, coagulase-negative staphylococcus, *Pseudomonas aeruginosa*, fungi, and HACEK (*Haemophilus*, *Actinobacillus actinomycetemcomitans*, *Cardiobacterium hominis*, *Eikenella corrodens* and *Kingella kingae*), do occur, and tend to be associated with poorer outcomes.

Antibiotic treatment should start immediately after blood cultures are obtained. First line treatment is parenteral bactericidal antibiotics for 4–6 weeks. For persistent and invasive infections, refractory heart failure and prosthetic valves, surgical intervention is indicated. However, the high recurrence rate caused by continuing IDU and medical non-compliance inevitably raises the ethical consideration of offering valve surgery to this patient population.

## Case report

A 45-year-old African American male with a medical history significant for active IDU, hypertension, type II diabetes mellitus, hepatitis C, history of osteomyelitis and cerebrovascular accident was brought to emergency room for mental status changes. He was found to be in respiratory failure and was subsequently intubated in the ER.

**Fig. 1:**
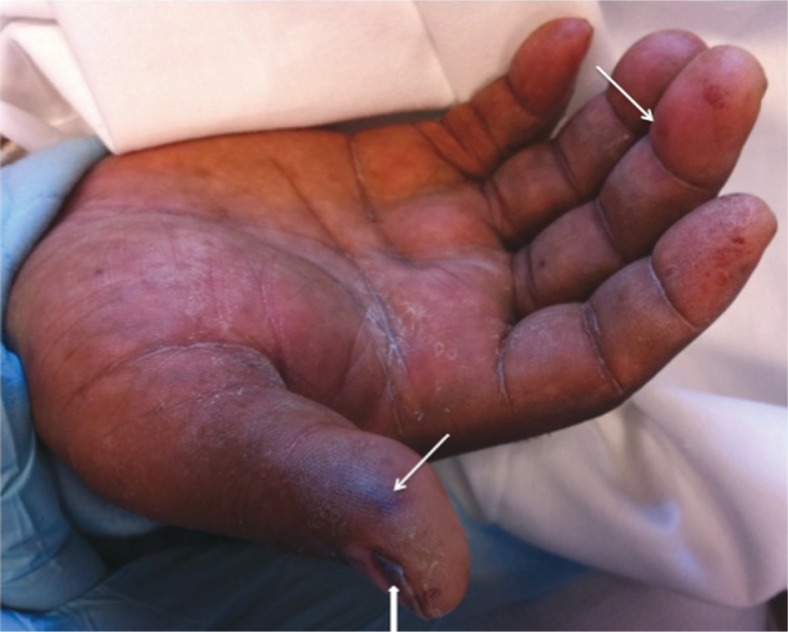
Janeway Lesion showing as painless, macular, hemorrhagic, irregularly-shaped lesions on patient's palm. Two pronounced lesions are seen at thumb and middle finger. Subungual splinter hemorrhages (arrowhead) are seen at the nail bed of thumb.

Physical examination on presentation was significant for temperature of 39.3°C, controlled blood pressure, sinus tachycardia at a rate of 105 beats/min, and a respiratory rate of 28/min. Oxygen saturation was 98% on AC mode with 35% oxygen concentration. His pupils were pinpoint. The patient had multiple abscesses on the upper extremities at the sites of IV drug injection. Multiple pink, macular, irregular lesions were seen on the patient's right thumb and hand (Fig. 1).

Laboratory results included leukocytosis of 14.8 with 85.1% neutrophils, lactic acid 15.9, hyperglycemia 460 with negative ketones, and increased BUN/creatinine 24/2. HIV screening test was negative. Chest X-ray and head CT were initially unremarkable. CSF analysis revealed elevated protein 118 mg/dL, glucose 240 mg/dL with WBC 73 (segment 98%). However, CSF gram stain and culture were negative. Blood cultures were positive for MSSA.

The patient was admitted to the intensive care unit and antimicrobial treatment was initiated (ceftazidime 1 g IV q8h – later switched to ceftriaxone and vancomycin 1.5 g IV q12h). Transthoracic echocardiography (TTE) obtained on the day of admission did not reveal any detectable intracardiac vegetations. Despite the aggressive antibiotic treatment, the patient remained febrile. On the fifth day of his hospital stay, transesophageal echocardiography (TEE) was performed, and a 1.5 cm vegetation was found on the aortic valve. The antibiotic regimen was then changed to nafcillin (2 g IV q4h) and gentamicin (3 mg/kg qday for 5 days) for MSSA positive endocarditis. The patient, however, did not improve and 2 weeks after admission, had a cardiac arrest and died.

## Discussion

### Etiology

Similarly to most examples of IE, *Staphylococcus aureus* is the causative agent in majority of IE cases (60–70%) related to IDU and is mostly MSSA. However, less common infections including Methicillin resistant staphylococcus aureus (MRSA) (up to 26%), streptococci viridans (8–10%), enterococci (2–5%), streptococci bovis or other streptococci species (3%), coagulase-negative staphylococci (3%), and fungi (1–2%), do occur (6, 7). Other rare causes include *Pseudomonas aeruginosa*, HACEK organisms, *E. coli*, *Corynebacterium*, *Proteus mirabilis*, *Mycobacterium tuberculosis*, and *Bacteroides fragilis* (8). Approximately 5% of cases have negative blood cultures (9). Furthermore, these values are not static. Community and healthcare related MRSA infections are increasing in prevalence, and are becoming more common in IE (6, 10, 11, 23).

IDUs are prone to acquiring needle-borne infections, either from the skin, or contaminated injection equipment, drugs and solvents. Moreover, many IDUs are immunocompromised after being infected with HIV and/or hepatitis C virus (12).

The tricuspid valve is infected in 50–70% of IDU cases of IE. The mitral and aortic valves are involved in 20–30% of cases. The tricuspid valve may be more susceptible to heroin use, as heroin can cause an increase in pulmonary arterial pressure, creating more turbulence at the tricuspid valve. Substances such as cocaine and metamphetamines, on the other hand, increase systemic afterload, causing increased turbulence at the sites of the aortic and mitral valves. Therefore, any shifts in the incidence of right versus left-sided IE may reflect the availability of certain illicit substances (6, 10).

Clinical studies have shown the difference in prognosis between right-sided and left-sided IE. Compared to right-sided IE (mortality <5%), left-sided IE, especially when the aortic valve is involved, has worse outcomes (mortality 20–30%). IDU with HIV infection have an even greater chance of acquiring IE (odds ratio of 2.3 in CD4 > 350 and 8.31 in CD4 < 350) compared to patients who tested negative for HIV (13).

### Diagnosis

Diagnosis of IE at present relies on the modified Duke Criteria. The two major criteria are sustained bacteremia and involvement of the endocardium, which can be determined either by using an echocardiogram searching for vegetations, abscesses or new valvular regurgitation. The minor criteria include predisposing heart diseases, fever, vascular (for example, Janeway lesions or septic emboli) or immunologic (for example, positive rheumatoid factor, glomerulonephritis) phenomena, and microbiological or echocardiography evidence which does not meet major criteria.

The classic physical findings (Janeway lesions, Osler's nodes, Roth spots and splinter hemorrhages) are rarely seen, appearing only in 5–15% of IE patients. Instead the more common manifestations are the complications of advanced stage disease, including septic emboli and organ infarction.

Janeway lesions are painless, macular, hemorrhagic, irregularly-shaped lesions that occur on the palms and soles. The pink macular lesions on our patient's right hand, we believe, represented Janeway lesions (Fig. 1, arrow). Moreover, a pronounced subungual splinter hemorrhage was also seen at the patient's thumb (Fig. 1, arrowhead).

The sensitivity of blood cultures is over 90% if they are sent before administration of antibiotics. Culture-negative IE is mostly a consequence of prior antibiotic usage; however, infections by fastidious and atypical organisms, including *Bartonella*, *Coxiella*, *Legionella*, HACEK family, etc, are becoming increasingly common, especially in immunocompromised patients or patients with foreign bodies, such as prosthetic valves, central venous catheters or pacemakers (14). Diagnosis of culture negative IE is made by modified culture conditions, serology, molecular techniques or immunohistology of the surgically removed specimens.

When suspicious for IE, TTE is sensitive for detecting vegetations and identifying the affected valves. Overall the sensitivity is 60–70%, whereas it can detect tricuspid vegetations with higher sensitivity (80%) (15). However, TEE has better sensitivity and specificity when compared to TTE. The superior sensitivity of TEE has again been demonstrated by our patient. It should be obtained when the TTE study is negative and clinical suspicion is high, in all prosthetic valve IE, and in patients with invasive infections and complications. Apparently, TEE also has enhanced sensitivity in terms of detecting aortic valve vegetations (16). This also holds true in our patient.

### Treatment

Similar to other causative agents of IE, treatment of IE in IDU is still largely dependent on antimicrobial chemotherapy. The choice of antibiotics essentially depends on the likely microorganisms, involved valves and the types of injected drugs the patient has used. Since MSSA is the most common microorganism, narrow-spectrum beta-lactamase-resistant penicillin such as nafcillin or oxacillin (8–12 g/day) is the first line agent. Penicillin G (12–18 million units/day) is given for streptococci viridans and bovis (minimum inhibitory concentration (MIC) <0.5). Ampicillin (12 g/day) is the drug of choice for enterococci and streptococci with penicillin MIC > 0.5. It is still debatable whether addition of gentamicin is beneficial, especially in patients infected with MSSA or penicillin-susceptible streptococci. However, the synergistic effect of gentamicin with penicillins may be important in the eradication of MRSA or penicillin-resistant streptococci, as well as in IDU patients and patients with prosthetic valves. When it is used, gentamicin is normally only given during the first 3–5 days. The patient in our case report was given both nafcillin and a 5-day course of gentamicin after positive blood cultures for MSSA were confirmed.

For patients allergic to penicillin, cephalosporins such as cefazolin (6 g/day) or ceftriaxone (1–4 g/day) are generally used if the patient has no history of anaphylactic reactions. Vancomycin is used if the patient has a history of anaphylaxis.

If the etiological agents are MRSA, coagulase-negative staphylococci, *Pseudomonas aeruginosa*, fungi, or HACEK, the treatment is similar to IE in non-IDU patients. MRSA is treated with vancomycin; however, treatment failures of MRSA with vancomycin are becoming increasingly common. Agents such as daptomycin, linezolid and quinupristin-dalfopristin are suggested as alternatives in cases of vancomycin-intermediate MRSA. For treatment of other uncommon pathogens, please read references (7, 15, 17–20, 24).

In IDU-related IE, the duration of antibiotic therapy is normally 4–6 weeks. It has been demonstrated in some studies that a 2-week combined therapy of penicillinase-resistant penicillin plus aminoglycosides for non-complicated MSSA right-sided IE has similar efficacy to longer duration treatment. However, if the patient has left-sided or complicated right-sided IE, such as the presence of heart failure, acute respiratory failure, renal failure, empyema, systemic septic emboli or vegetation size >2 cm, the standard 4 to 6-week duration therapy must be used.

Surgical intervention is indicated if the patient has refractory heart failure, persistent or recurrent infections including bacteria and fungi, prosthetic valves, development of a ring abscess, fistulae or worsening conduction abnormalities. However, special considerations are involved in the surgical treatment of IE in the population of IDU. The likelihood of continuing parenteral drug misuse carries high risk of re-infection, overdose and other complications.

There has been a continuous debate as to whether it is ethical to withhold valve surgery in an active, noncompliant drug user. The first valve surgery is normally offered to those patients who are willing to undergo drug rehabilitation program and be compliant with treatment. However, any IDU who becomes non-compliant, relapses, and acquires a second episode of IE generally will not be offered further valve surgeries (21).

### Prognosis

The outcome of right-sided MSSA IE is good with a mortality rate of <5%. Left-sided IE, especially when the aortic valve is involved, and complicated IE are associated with worse outcomes, and have a mortality rate of >20–30%. Etiological agents also affect the prognosis. Gram negative bacilli and fungus tend to have poorer outcomes. Furthermore, severe sepsis, systemic septic embolization and multi-organ failure increase the risk of death. Compared to the general population, IDU have a more severe clinical course, including poorer cardiac function and a higher rate of systemic embolization (notably CNS embolization), which are usually the causes of death. Moreover, higher rate of recurrence is expected in this patient population. Thus, aggressive medical and surgical treatments are required (22).
